# Ivermectin treatment response in two rural villages with a high prevalence of onchocerciasis and epilepsy, Mahenge Tanzania

**DOI:** 10.4314/ahs.v22i3.65

**Published:** 2022-09

**Authors:** Dan Bhwana, Bruno P Mmbando, Alfred Dusabimana, Athanas Mhina, Daniel P Challe, Joseph N Siewe Fodjo, Williams H Makunde, Robert Colebunders

**Affiliations:** 1 National Institute for Medical Research, Tanga Research Centre, Tanga, Tanzania; 2 Global Health Institute, University of Antwerp, Antwerp, Belgium

**Keywords:** Onchocerciasis, ivermectin, treatment response, epilepsy, Tanzania

## Abstract

**Background:**

Despite 20 years of ivermectin mass distribution in the Mahenge area, Tanzania, the prevalence of onchocerciasis and epilepsy has remained high in rural villages.

**Objectives:**

We investigated the efficacy of ivermectin in reducing Onchocerca volvulus microfilariae and predictors for parasitic load following ivermectin treatment in persons with (PWE) and without epilepsy (PWOE).

**Methods:**

Between April and September 2019, 50 PWE and 160 randomly selected PWOE from Msogezi and Mdindo villages participated in a follow-up study. Skin snips were obtained pre (baseline) and three months post-ivermectin treatment.

**Results:**

The overall prevalence of *O. volvulus* positive skin snips at baseline was 49% (103/210), with no significant difference between PWE (58.0%) and PWOE (46.3%); p=0.197. The overall mean microfilarial density was significantly higher at baseline 1.45(95%CI:0.98–2.04)) than three-month post-ivermectin treatment (0.23(95%CI:0.11–0.37), p<0.001. Three months after ivermectin, the microfilarial density had decreased by ≥80% in 54 (81.8%, 95%CI: 72.3–91.4) of the 66 individuals with positive skin snips at baseline. High microfilarial density at baseline was the only significant predictor associated with higher microfilarial density in the post-ivermectin skin snips.

**Conclusion:**

Our study reports a decrease in microfilarial density following ivermectin treatment in most individuals. Optimizing ivermectin coverage will address the ongoing onchocerciasis transmission in Mahenge.

## Introduction

Onchocerciasis, commonly referred to as river blindness, is caused by the nematode Onchocerca volvulus, and is transmitted by a black fly of the genus Simulium. It is estimated that 20.9 million people are infected worldwide, and 99% of them are living in sub-Saharan Africa 1. Adult worms in nodules can live for more than a decade, during which the female worm may release 1300–1900 microfilariae (mf) per day 2. In the skin, mf are found in the sub-epidermis, where they can cause severe itching and disfiguring skin lesions. They may also migrate to the anterior chamber of the eye where they can cause blindness^2^. In heavily infected persons, more than 100 million mf may be present; each of which can live for up to two years 2. The World Health Organization (WHO) enlisted onchocerciasis as a priority disease targeted for elimination by 2025 using a community-directed treatment with ivermectin (CDTI) strategy 3,4.

Ivermectin has a microfilaricidal effect which substantially reduces microfilarial densities in the skin and eyes within days to weeks 2. A systematic review and meta-analysis of 26 studies, showed that a single dose of ivermectin resulted in a marked decrease in mf density within 24 hours; furthermore, approximately 95% of the mf were cleared within the first week and 99% thereof after 1 to 2 months 5. Since ivermectin does not kill the adult worms but temporarily blocks the release of mf from the female adult worms, microfilarial repopulation of the skin starts at a slow rate approximately three months after ivermectin treatment 6. However, skin mf densities are expected to remain below 20% of their pre-ivermectin levels for at least 10 months after a single dose of ivermectin 5.

Currently, several epidemiological studies have shown a strong relationship between onchocerciasis and epilepsy, including nodding syndrome (NS) 7–16. In a meta-analysis investigating the association between onchocerciasis and epilepsy, it was found that for each 10% increase in onchocerciasis prevalence, the prevalence of epilepsy increases on average, by 0.4% 17. Recent observations in northern Uganda 18 and Cameroon 13 suggest that onchocerciasis control through mass drug administration (MDA) using ivermectin may decrease the incidence of NS and other forms of onchocerciasis-associated epilepsy (OAE).

In January 2017, a house to house epilepsy survey in the Mahenge area of Tanzania documented an epilepsy prevalence of 1.5% in sub-urban and 3.5% in rural villages, Mdindo and Msogezi 11. Moreover, the prevalence of anti-onchocercal antibodies, using the OV16 rapid test 19, among children aged 6–10 years was 38% in Mdindo and 42% in Msogezi, suggesting ongoing transmission of onchocerciasis^11^. While such high transmission rates were unexpected after 20 years of CDTI, possible explanations include low ivermectin coverage 20, and/or reduced effectiveness of ivermectin in reducing *O. volvulus* mf density. Therefore, we returned to these two rural villages and carried out a study to investigate the efficacy of ivermectin in reducing *O. volvulus* mf in persons with and without epilepsy, as well as to identify factors associated with detectable *O. volvulus* mf, 3 months after ivermectin treatment.

## Materials and Methods

### Study design, sites, and participants

This was a longitudinal study conducted in the two rural villages of Msogezi and Mdindo in Mahenge, between April and September 2019. The details of the study area have been extensively described in our previous publication^11^. In brief, the Mahenge area extends into a mountainous area above the Kilombero river valley in which most of the northern part of Ulanga district villages are located. The area contains fast flowing rivers with rapids which are excellent breeding sites for black flies. The Ulanga district has 21 wards and 59 registered villages that are considered endemic for onchocerciasis and epilepsy. Using a community-based approach, we invited persons with epilepsy (PWE), identified during the epilepsy surveys conducted in January 2017 11,21, to participate in this new study. Among PWE identified in these surveys, 77.9% of them met the OAE case definition criteria, defined as a previously healthy person who had developed epilepsy without an obvious cause between the ages of 3 and 18 years 22. Similarly, persons without epilepsy (PWOE) aged >6 years were randomly selected from the database of the aforementioned surveys, and also invited to participate. Informed consent was obtained from all participants, directly from the PWE (for adults) or from their parents/care givers (for children) before their enrolment into the study. For children older than 12 years, their assent was also obtained. Participants were enrolled in the study between April and May 2019; 7 months after the previous round of community direct treatment with ivermectin (CDTI) in 2018 (considered as baseline). They were skin snipped just before receiving ivermectin during CDTI in May and June 2019. The ivermectin dose was height-dependent and its administration was directly observed under CDTI program. Skin snipping was repeated three months after ivermectin treatment. Clinical examination information was collected using a paper based questionnaire. The questionnaire contained three sections; a section about 1) social-demographic characteristics; 2) epilepsy related information such as type of seizures, frequency of seizures and clinical examination; 3) onchocerciasis related information including history of itching, past ivermectin use and skin snip testing results.

### Skin snip collection and processing

Two skin snips were obtained from each posterior iliac crest of eligible participants using the Holt-type punch. One punch was used per subject and punches were sterilised using steam under pressure before using on other subjects. Moreover, to prevent infection, the skin was cleaned with 70% alcohol before snipping. Skin snips were placed in different wells of a micro titre plate containing 3 drops of normal saline solution and incubated for 24 hours at room temperature to allow the mf to emerge into the fluid. After the incubation period, mf in the solution were counted microscopically by a trained laboratory technician using x10 objective. For each person, the two skin snips were weighed using electronic scales and average was obtained. Mf density was expressed in counts per milligram of skin snip.

### Data management

Completed data collection forms were cross-checked on a daily basis at the field site to ensure completeness and accuracy of data collected. Data were entered into a Microsoft Access database by experienced data entry clerks and checked by a data coordinator for completeness and quality by reviewing 5% of all records selected at random. Skipping patterns and range checks were implemented in the database to minimise chances of error. All queries raised during data entry and cleaning were sent back to the field coordinator for resolution.

### Statistical analysis

Continuous variables were presented as means with 95% confidence intervals and as medians with inter-quartile ranges (IQR), compared using the Mann-Whitney U test. Proportions were expressed as percentages and compared using Chi-squared tests or Z-test where applicable. The mf density for each participant was expressed as the arithmetic mean from both skin snips. The community mf load (CMFL) for each village was calculated as the geometric mean mf density per skin snip in persons aged 20 years and older. The arbitrary value of 1 was added in this calculation to control for persons with no (zero) mf in their skin snips. Pre- and post-mf density were compared using Wilcoxon signed-rank test. Ivermectin response rate was calculated as the proportional change of mf density for all individuals who were seen before and three months after CDTI, and had positive skin snips at baseline. Given that we expect less than 20% of the baseline *O. volvulus* parasitic load in an individual treated with ivermectin, any mf density reduction below 80% three months after ivermectin treatment was considered as a sub-optimal response to ivermectin.

A multiple logistic regression models was used to identify determinants of sub-optimal response to ivermectin. Predictors of post-ivermectin mf density in participants who initially had positive skin snips were determined using a negative binomial model. Data were analysed using SAS 9.4 (SAS institute Inc.) and R version 3.6.1. A p-value less than 0.05 was deemed significant.

## Results

### Baseline data

A total of 210 participants were enrolled in the study, of which 50 (23.8%) were PWE and 104 (49.5%) were females (Table 1). Among the PWE, 32 (64%) were from Msogezi and 18 (36%) from Mdindo village. The overall median age of the participants was 31 years (IQR: 15–48) while that of PWE was 30.0 (IQR: 24.0–39.0). The overall prevalence of *O. volvulus* mf was 49%, and this tended to be higher among PWE (58.0%) compared to PWOE (46.3%), although the difference was not statistically significant (p=0.195). The prevalence of mf was significantly higher among participants from Mdindo village (64.8%) compared to Msogezi (35.2%), χ2=9.57, p= 0.002. The CMFL was also higher in Mdindo village (3.8mf/mg) compared to Msogezi (1.9 mf/mg), z = 3.2, p = 0.001.

**Table 1:** Characteristics of the study participants before the intake of ivermectin

	Overall N = 210	PWE n = 50	PWOE n = 160	P value*
**Socio-demographics**				
Age: median (IQR)	31 (15–48)	30.0 (24.0–39.0)^a^	32.0 (14.0–51.0)^b^	0.544
Female gender: n (%)	104 (49.5)	24 (48.0)	80 (50.0)	0.872
Study village: Mdindo, n (%)	74 (35.2)	18 (36.0)	56 (35.0)	0.927
Msogezi, n (%)	136 (64.8)	32 (64.0)	104 (65.0)	
**Anthropometrics**				
Height in cm: median (IQR)	152.0 (140.0–159.0)	152.5 (143.0–156.8)	152.0 (139.9–160.0)	0.765
Body mass index	19.6 (17.1–22.1)	20.4 (18.4–22.0)	19.5 (16.9–22.4)	0.380
Weight in Kg: median (IQR)	46.0 (37.3–53.0)	46.0 (40.3–52.0)	46.8 (34.8–53.1)	0.966
**Onchocerciasis**				
Positive skin snip: n (%)	103 (49.0)	29 (58.0)	74 (46.3)	0.195
Pre-IVM mf density for all participants: median (IQR)	0.0 (0.0–2.7)	0.6 (0.0–4.4)	0.0 (0.0–1.9)	0.315
Pre-IVM mf density for participants with positive skin snip: median (IQR)	3.6 (1.7–13.3)	3.0 (1.7–11.3)	4.24 (1.7–15.6)	0.886
Palpable nodules: n (%)	19 (9.0)	8 (16.0)	11 (6.9)	0.085
Itching: n (%)	51 (32.1)	9 (24.3)	42 (34.4)	0.316
Ivermectin use in 2018: n (%)	159 (75.7)	37 (74.0)	118 (75.6)	0.706

a 1 missing observation;

b 7 missing observations

IQR: interquartile range, mf: Microfilarial; IVM: ivermectin, NA: Not applicable

* Mann Whitney U test for continuous variables, Chi-squared test for categorical variables (Fisher exact test if expected values < 5)

**Figure 2:**
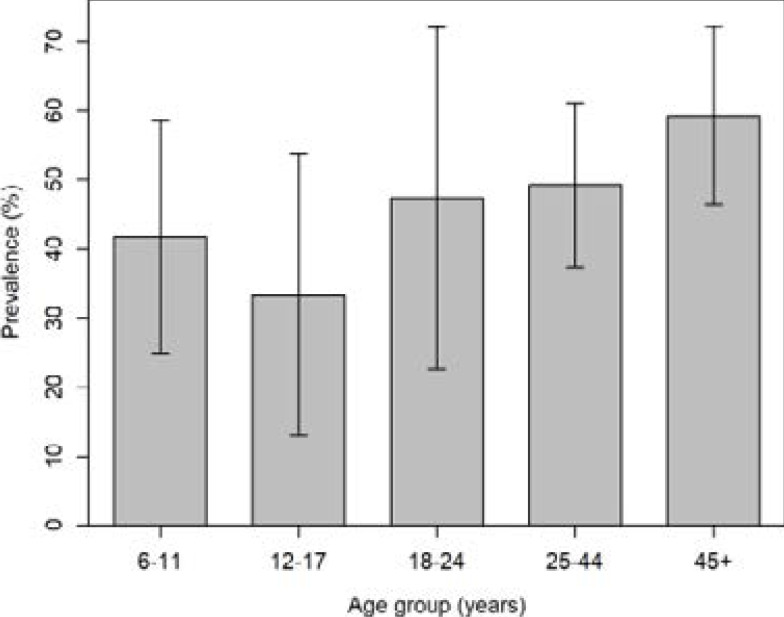
Prevalence of positive skin snip (bars) with 95%CI (line segments) by age group

The proportion of individuals with positive skin snips increased with increasing age, χ2 trend= 4.29, p=0.038. However, the proportion of positive skin snips in the youngest age group (6–11 years) was slightly higher (41.7%, 95%CI: 24.8–58.6) than in the subsequent age group (33.3%, 95%CI: 13.0–53.7), Figure 2.

### Effect of ivermectin on mf density among *O. volvulus* infected PWOE and PWE

Of the 210 study participants examined at baseline, 163 (77.6%) were available for a follow-up skin snip sample three months post-ivermectin. The main reasons for not participating in the post-ivermectin skin snip testing included refusal due to discomfort caused by the initial skin snipping, and being away from the study villages during the second skin snipping period. Of the 163 participants with a follow up skin snip sample, 76 (46.6%) had a positive skin snip at baseline compared to 29 (17.8%) post-ivermectin, p< 0.001. The proportion of individuals who took ivermectin during the 2018 CDTI increased significantly from 75.5% at the baseline survey to 92.0% during the post ivermectin survey, (Mcnemar's-χ2 =18.7, p<0.001), (Table 2). Considering only PWOE and PWE who took ivermectin treatment, >80% decrease in mf density was observed in 54/66 (81.8% [95% CI: 72.3–91.4]) participants who were infected at baseline. However, only 14 out of 19 PWE (73.7% [95% CI: 51.9–95.5]) attained the threshold of >80% decrease in mf density. There was no difference in frequency of seizures at baseline and post ivermectin, p=0.480 (Table 2).

**Table 2:** Response of ivermectin on mf density in all 163 participants with both baseline and three months post-ivermectin skin snip results

	Skin snip survey		
		
Characteristics	Baseline	Post-ivermectin	P-value*
**All participants (n=163)**			
Took ivermectin in previous CDTI round, n (%)	123 (75.5)	150 (92.0)	<0.001
Positive skin snips: n (%)	76 (46.6)	29 (17.8)	<0.001
Mf density: median (IQR)	0.0 (0.0–2.8)	0.0 (0.0–0.0)	<0.001
Mf density: mean (95%CI), n=162	1.45 (0.98–2.04)	0.23 (0.11–0.37)	<0.001
>80% reduction in mf density (only for participants with positive skin snip at baseline): n (% [95% CI])	NA	54/66 (81.8 [72.3–91.4])^c^	NA
**PWE only (n=42)**			
Positive skin snips: n (%)	22 (52.4)	13 (30.9)	0.020
Mf density: median (IQR)	0.26 (0.0–3.78)	0.0 (0.0–0.23)	<0.001
Mf density: mean (95%CI)	1.73 (0.76–3.24)	0.22 (0.07–0.40)	<0.001
>80% reduction in mf density (only for PWE with positive skin snip at baseline): n (% [95% CI])	NA	14/19 (73.7 [51.9–95.5])^d^	NA
Experienced a seizure in the last week, n (%)	8/32 (25)^e^	10/32 (31.2)^e^	0.480
Seizures per month: median (IQR)	0.0 (0.0–2.0)	0.0 (0.0–0.5)	0.241

*Mcnemar's test, *Wilcoxon signed-rank test

c 10 missing observations post-ivermectin

d 3 missing observations

e 10 missing observations post-ivermectin

IQR= interquartile range, Mf=microfilarial

Overall, the geometric mean mf density decreased from 1.45 mf/mg (95% CI: 0.98 - 2.04) at baseline to 0.23 mf/mg (95% CI: 0.11- 0.37) 3 months post-ivermectin, p <0.001. Majority (71.0%) of 76 people who tested skin snip positive at baseline tested negative three months post-ivermectin, while among those who had negative skin snips (n=87), 7 (8.0%) of them tested positive three months post the ivermectin, giving an OR of 7.71 (95%CI: 3.50–20.10), McNemar's P < 0.001.

Skin snips positivity rate among PWE decreased from 52.4% (22/42 PWE) at baseline to 30.9% (13/42 PWE) three months post ivermectin; p=0.020. Of the 22 PWE who were positive at baseline, 12 (54.5%) had no detectable mf in their skin snips post-ivermectin, while only 3 (15%) out of 20 PWE with no mf detected at baseline were detected with mf post-ivermectin, giving an OR of 4.0 (95%CI: 1.08–22.09), McNemar's p = 0.020; this means the rate of converting from positive to negative after CDTi was four folds compared to that of changing from negative to positive.

Although in univariate analysis, three factors (being a PWE, village of residence and pre-ivermectin mf density) were found to be associated with persistence of mf three months post ivermectin treatment, in multivariate analysis only being a PWE (OR=2.65, p=0.033) and pre-ivermectin mf density (OR=1.54, p=0.003) remained significant (Table 3). Self-reported history of ivermectin use during the past years was not associated with skin snip positivity following ivermectin treatment in this study.

**Table 3:** Factors associated with positive skin snips three months post-ivermectin

Covariates	Univariate		Multivariate	
		
	OR (95%CI)	P-value	OR (95%CI)	P-value
Sex (Female)	0.95 (0.43–2.12)	0.902		
Age (in years)	1.00 (0.98–1.02)	0.675		
Being a PWE	2.56 (1.10–5.95)	0.030	2.65 (1.08–6.51)	0.033
Village of residence: Mdindo	1			
Msogezi	0.43 (0.19–0.96)	0.040	0.59 (0.24–1.45)	0.249
BMI – Normal or overweight (≥ 18.5)	1			
– Underweight (< 18.5)	0.80 (0.35–1.80)	0.582		
Pre-ivermectin mf density*	1.62 (1.24–2.11)	<0.001	1.54 (1.16–2.03)	0.003
Never used ivermectin	1.07 (0.29–4.04)	0.916		
Did not take ivermectin in 2018 (most recent CDTI before this study)	1.61 (0.48–5.41)	0.439		

* Log-transformed

### Predictors of mf density 3 months after ivermectin use

The mf density at three months post-treatment ranged from 0.0 to 100 mf/mg. In a univariate negative binomial regression model, both higher pre-ivermectin mf-density (IR= 1.77, 95% CI: 1.27–2.46, p=0.001) and village of residence (Msogezi village; IR=0.33, 95%CI: 0.12–0.91, p=0.032) were the variables significantly associated with mf density three months after ivermectin treatment. However in the multivariate model, only pre-ivermectin mf density remained significant (IR=1.65, 95%CI: 1.18–2.31, p=0.003), (Table 4).

**Table 4:** Factors associated with mf density three months post ivermectin use

	Univariate		Multivariate	
		
Covariates	IR (95%CI)	P-value	IR (95%CI)	P-value
Sex (Female)	0.72 (.26 – 1.98)	0.528		
Age (in years)	1.01 (0.99 – 1.04)	0.233		
Being a PWE	0.997 (0.34 – 2.97)	0.996		
Village of residence: Msogezi	0.34 (0.12 – 0.91)	0.032	0.577 (0.20–1.67)	0.310
BMI – Underweight (< 18.5)	0.50 (0.18 – 1.38)	0.180		
Pre-ivermectin mf density*	1.77 (1.27–2.46)	0.001	1.65 (1.18 – 2.31)	0.003

* Log-transformed

## Discussion

This study aimed to determine the effect of ivermectin treatment on mf density in *O. volvulus* infected PWE and PWOE in Mahenge and to identify risk factors for sub-optimal ivermectin response. Three months after receiving ivermectin, only 18% of participants had < 80% reduction of the pre-ivermectin mf density, suggesting that sub-optimal response to ivermectin is not frequent in this population. The reason for the reduced efficacy of ivermectin in a few participants remains unclear and warrants further investigations. Reasons to consider include: false self-reported use ivermectin in the recent CDTI, variation in distribution of mf in the sampled snips, as well as differences in incubation periods during laboratory analyses 23.

The high overall prevalence of skin snips positive for *O. volvulus* mf in all individuals (49%) as well as in children aged 6–11 years (41.7%) confirms a high burden of onchocerciasis with high on-going transmission in the Mahenge area. In 2017, we found almost a similar prevalence of onchocerciasis by using OV16 antibodies tests in children aged 6–10 years 11, a pattern which was also depicted by an entomological study in 2016 in the same area 24.

A slightly higher percentage of PWE (58%) compared to PWOE (46%) presented with a positive skin snip at baseline; however, their mf densities were very low. Although this excess mf prevalence of 12% among PWE is statistically non-significant (due to small sample size), it does not imply that there is no association between onchocerciasis and epilepsy in the rural villages in the Mahenge area. Indeed, in settings where CDTI has been implemented for many years, the interpretation of these results is difficult because past ivermectin intake among PWE and PWOE may have been different. It is possible that at epilepsy onset, PWE were more often *O. volvulus* infected and with a higher mf load than PWOE. Although a similar percentage of PWE and PWOE reported ivermectin intake in 2018, data is lacking regarding individual ivermectin intake before and at the moment of the onset of the epilepsy. In several case-control studies in onchocerciasis-endemic regions where CDTI has been implemented for some time, no difference in skin snip positivity was observed between PWE and PWOE 25. In contrast, in matched case-control studies in ivermectin-naïve populations in an onchocerciasis-endemic region in Cameroon^26^, and in the Logo health zone in Ituri, Democratic Republic of Congo (DRC) 27 PWE were more often found to be skin snip positive and to have a higher mf load than healthy village controls. In the present study, the logistic regression revealed a significant association between the persistence of *O. volvulus* microfiladermia and having epilepsy, as PWE had 2.6 folds higher odds of presenting a positive skin snip three months post ivermectin when compared to PWOE. An explanation could be the potential decrease of ivermectin drug levels in the serum because of the anti-epileptic drugs affecting the P-glycoprotein (MDR1) transporter which plays a role in the intestinal elimination of the ivermectin^28^.

We did not observe differences in frequency of seizures in PWE at the baseline and post-ivermectin intake. There is anecdotal evidence that ivermectin in *O. volvulus* infected PWE may decrease the frequency of seizures 29. Moreover, a recent randomised clinical trial in DRC showed that *O. volvulus* infected PWE who received multiple doses of ivermectin were more likely to be seizure-free during the last four months of a one year trial than *O. volvulus* PWE who received only one dose of ivermectin 30. The small sample size of our study and the low mf density at baseline, most likely explains why a beneficial effect of ivermectin was not observed.

Our results suggest that ivermectin is still a potent microfilaricide in the rural villages of the Mahenge area. This is similar to observations from Equatorial Guinea 31 but different from the situation in Ghana where a sub-optimal ivermectin treatment response was reported 6,32,33.

Therefore the high *O. volvulus* transmission that was observed in the rural villages of Mahenge is probably the result of sub-optimal ivermectin coverage in the past which could be due to poor programme supervision and poor compliance of communities with the CDTI programme; underlying causes could include lack of knowledge, poor advocacy and low motivation among drug distributors 11. However, our study also has several limitations. The number of PWE who agreed to participate was small, thereby decreasing the statistical power of our analysis. Moreover in this study, no other laboratory examinations apart from skin snips were performed to exclude other causes of epilepsy, such as genetic, or other parasitic causes than onchocerciasis. As 77.9% of the PWE met the OAE criteria, we estimate that in a large proportion of the PWE the epilepsy was related to onchocerciasis. Moreover, the higher prevalence of epilepsy in rural villages in Mahenge with higher ongoing *O. volvulus* transmission, compared with sub-urban Mahenge villages, suggests that *O. volvulus* infection was the main cause of the high epilepsy prevalence in our study population^11^. However, we cannot exclude the presence of neurocysticercosis in the region but Taenia solium transmission may be low because pigs are generally kept and fed in small elevated huts preventing their contact with human stools^34^.

## Conclusion

Onchocerciasis transmission is ongoing in rural villages of Mahenge and the response of the parasite to ivermectin is satisfactory. However, the interpretation of our study findings should be done with caution. Indeed, the number of those with <80% mf reduction was rather low but it is not clear whether these were suboptimal responses. Nevertheless, there is need to strengthen the onchocerciasis elimination programme in Mahenge in order to optimize coverage, thereby accelerating elimination prospects. In that light, the national neglected tropical diseases programme initiated a biannual CDTI programme in Mahenge as from 2019. We anticipate that a follow-up of this intervention should eventually reveal a diminishing incidence of both onchocerciasis and onchocerciasis-associated epilepsy in the Mahenge area.

## Figures and Tables

**Figure 1:**
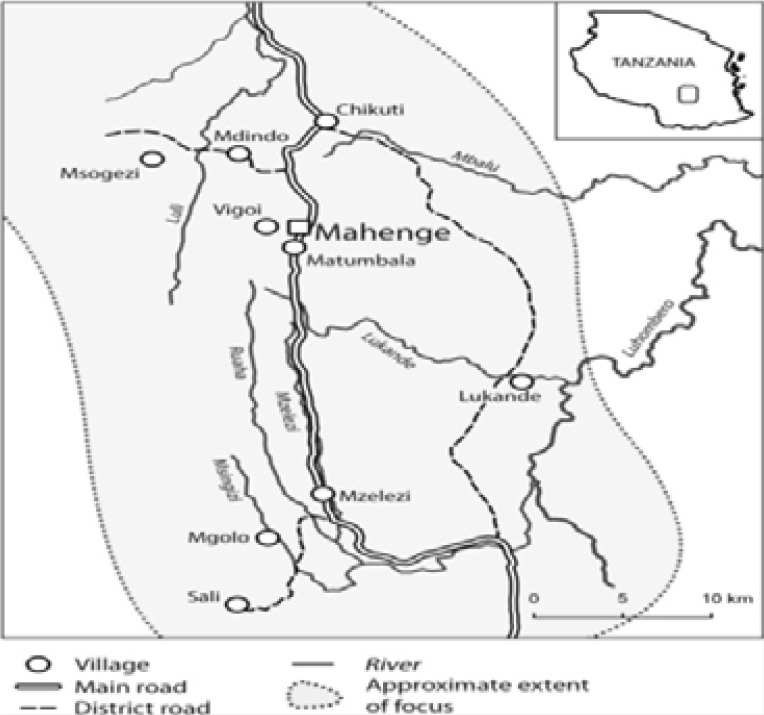
Map of the Mahenge area (Figure by A Hendy^24^).
